# Decline in the mental health of nurses across the globe during COVID-19: A systematic review and meta-analysis

**DOI:** 10.7189/jogh.11.05009

**Published:** 2021-04-10

**Authors:** Abin Varghese, Gigini George, Sharat V Kondaguli, Abdallah Y Naser, Deepika C Khakha, Rajni Chatterji

**Affiliations:** 1 Bhopal Nursing College, Bhopal Memorial Hospital and Research Centre, ICMR, Ministry of Health & Family Welfare, Government of India, Bhopal, Madhya Pradesh, India; 2 Faculty of Pharmacy, Department of Applied Pharmaceutical Sciences and Clinical Pharmacy, Isra University, Amman, Jordan; 3 College of Nursing, All India Institute of Medical Sciences, Ministry of Health and Family Welfare, Government of India, New Delhi, India; 4 Department of Psychiatry, Bhopal Memorial Hospital and Research Centre, ICMR, Ministry of Health & Family Welfare, Government of India, Bhopal, Madhya Pradesh, India

## Abstract

**Background:**

Nurses represent the major proportion of frontline health care professionals delivering 24/7 services to patients with an increased vulnerability towards COVID-19 infection. Mental health issues among nurses during the COVID-19 pandemic are poorly reported across the globe. Henceforth, a systematic review and meta-analysis was performed to explore the prevalence and determinants of mental health outcomes (anxiety, stress, depression, PTSD, insomnia) among nurses across the globe due to the COVID-19.

**Methods:**

A PRISMA compliant systematic review (PROSPERO-CRD 42020204120) was carried out to identify articles from multiple databases reporting the prevalence of mental health outcomes among nurses. Proportion random effect analysis, *I^2^* statistic, quality assessment, and sensitivity analysis were carried out.

**Results:**

Pooled data on mental health outcomes were generated from 25 cross-sectional studies: 32% anxiety (95% confidence interval (CI) = 21%-44%, n (number of studies) = 21, N (sample size) = 13 641), 40.6% stress (95% CI = 25.4%-56.8%, n = 10, N = 4204), 32% depression (95% CI = 21%-44%, n = 17, N = 12 294), 18.6% PTSD (95% CI = 4.8%-38%, n = 3, N = 638), 38.3% insomnia (95% CI = 5.8%-78.6%, n = 2, N = 261) and significant risk factors for mental ailments includes; caring for COVID-19 patients, being a female, low self-efficacy, resilience, social support and having physical symptoms (sore-throat, breathlessness, cough, lethargy, myalgia, fever).

**Conclusion:**

The study results highlighted a higher proportion of poor mental health outcomes namely, anxiety, stress, depression, PTSD and insomnia among nurses from different parts of the world. Poor mental health outcomes among nurses warrants the need to implement proactive psychological interventions to deter the collapse of health care systems in responding to the pandemic and in particular all possible efforts should be undertaken to mitigate the risk factors. Health care organizations should provide support to nurses with sufficient flexibility. The disaster preparedness plan envisaged by nations should have provisions to address the mental health of nurses. Greater investment in addressing the global shortage of nurses should be given priority in national health policies. Attractive salary packages should be offered to nurses to prevent their emigration from low- and middle-income countries (LMICs).

**Registration:**

PROSPERO (CRD42020204120)

Coronavirus disease (COVID-19) or Severe Acute Respiratory Syndrome (SARS) CoV-2 has created havoc amongst people throughout the globe. Because of its greater infectivity, this pandemic has traversed various parts of the world at an unprecedented stride. The World Health Organization (WHO) has recorded more than 61.8 million cases and 1.4 million deaths as of 1st December 2020 [1]. As a result, health care facilities are overwhelmed with the patient load and strife to appease the demands of the population, placing immense strain on the frontline health care staff in exchange. Furthermore, a significant proportion of the frontline health care workers constitute nurses, providing round the clock services to patients with an increased vulnerability in getting infected [2]. Additionally, nurses’ workplace environment and work conditions put them at a higher risk of getting infected by patients, which can lead to mental health problems. Previous respiratory infections of this century such as SARS, Middle East Respiratory Syndrome (MERS), Ebola have demonstrated its psychological ramifications on nurses, manifested as stress, anxiety, depression, hostility, somatization and fear [3-6]. Furthermore, a higher prevalence of mental ailments has been reported among nurses compared to physicians and other health care workers during those infections [7,8]. Likewise, COVID-19 has also impacted the psychological health of nurses which is apparent from the multiple cross-sectional studies carried out across the various parts of the globe [9-13]. Moreover, nurses from Wuhan reported severe level of anxiety and depression than other frontline health care workers during the initial phase of COVID-19 [14]. Additionally, negative health outcomes are expected to be more common among health care providers during the COVID-19 pandemic compared to previous ones (SARS and MERS), this could be attributed due to multiple factors such as the shortage of personal protective equipment in some countries, increased working load, and having unexperienced clinical staff in coping with this new clinical situation and its associated new clinical guidelines [15]. According to WHO, the COVID-19 pandemic is likely to have both long and short-term influences on mental health. Due to the importance of the mental health implications of the pandemic, WHO [16] reported a list of considerations for the mental well-being of high-risk groups especially health care providers. A previous systematic review on factors that affects the psychological well-being of health care providers has identified the following factors to be highly influencing: having appropriate training and readiness, being at higher risk of infection due to work conditions, job stress, quarantine, perceived risk, and poor organizational support [17]. Preserving both the mental state and the medical conditions of health care providers who accept significant responsibility for treating patients with coronavirus infections is critical to sustaining the quality of appropriate health care services. Therefore, it is imperative to address the psychological impact of COVID-19 on nurses as the current priority. The scrutiny of literature did not yield any systematic review or meta-analysis addressing the current review question or inclusion criteria. Moreover, WHO [18] endorses an expeditious review of pragmatic substantiation to aid policymakers to frame recommendations that may augment the optimal response of health care systems. We hypothesized that nurses would suffer from poor mental health outcomes with numerous factors influencing them. Consequently, this meta-analysis was carried out to synthesize and present current evidence as; the pooled prevalence of anxiety, stress, depression, posttraumatic stress disorder, insomnia and to explore significant factors that are associated with the onset of mental ailments among nurses working in COVID-19 and non-COVID-19 centres during this pandemic and to determine the variations in the prevalence rates for multiple mental health outcomes across different regions of the world.

## METHODS

A primary search of the Joanna Briggs Institute Database of Systematic Reviews and Implementation Reports, the Cochrane Database of Systematic Reviews, PROSPERO and MEDLINE was performed before the registration of protocol to identify systematic reviews and meta-analysis that address the prevalence of mental health outcomes among nurses.

### Systematic review protocol registration

The protocol for this systematic review and meta-analysis has been registered at PROSPERO International Prospective Register of Systematic Reviews-CRD42020204120.

### Ethical approval

Ethical permission was not obtained for the study since we used published data that has already been ethically approved.

### Search process

We conducted the systematic review and meta-analysis following the preferred reporting items for systematic review (Table S1 in the **Online Supplementary Document**) and meta-analysis [19]. Original articles published from 11/03/2020 were searched in the databases; PUBMED, Web of Science Core Collection, MEDLINE, Psych Info, Nursing and Allied Health Database, Science Direct, Corona Virus Research Database and Google Scholar. Moreover, the preprint versions published in Medrxiv and SSRN servers were also included. Furthermore, we screened the references of relevant articles to retrieve more potential articles. The final search for all databases was completed on October 5, 2020. Multiple keywords (MeSH and free text word synonyms) such as nursing staff, COVID-19, mental health, severe acute respiratory syndrome coronavirus 2, depression, anxiety, stress was used individually or in combination with Boolean operators across several databases. We also have included, a detailed search strategy **(**Appendix S1 in the **Online Supplementary Document****).**

### Eligibility criteria

**Inclusion Criteria:** Published articles that fulfilled the following criteria were included: i) Population (P): assessed nurses who were working in hospitals in any country; ii) Intervention/Exposure(I): Intervention was not applicable, but included studies that analyzed the prevalence of mental health outcomes using validated instruments due to COVID-19 pandemic; iii) Comparator/control (C): No comparator or controls were applicable as we screened for epidemiologic studies reporting prevalence; iv) Outcomes (O): Prevalence of anxiety, stress, depression, PTSD, insomnia, significant risk factors for mental ailments; and iv) published articles in English language.

**Exclusion criteria:** We excluded studies with the following characteristics: i) Did not give an aggregate prevalence of anxiety, stress, depression, PTSD and insomnia even after contacting the corresponding author; ii) studies reporting the combined prevalence of mental health outcomes along with other health care professionals; iii) qualitative studies, systematic review, meta-analysis, case reports, case series and non-accessible full-text articles; and iv) studies with small sample size (N<40).

The titles and abstracts of studies were independently reviewed by two authors (AV, GG) for eligibility. Full texts of eligible studies were reviewed. Any disagreements between the two reviewers were solved by the third author (SV) by discussion and mutual consensus. Further RAYYAN QCRI was used to detect duplicates and search strategy management [20].

### Data extraction and appraisal of study quality

The following information was extracted from all included studies: author, month and year of publication, country, socio-demographic characteristics (sample size, marital status, gender proportion), the instrument used, prevalence and significant risk factors of mental health outcomes. The methodological quality of the included studies was evaluated using the Loney criteria which is a widely used tool to assess observational studies estimating the prevalence of health-related problems [21]. This tool consists of eight items which include: (1) random sample or whole population (2) unbiased sampling frame (3) adequate sample size (4) standard measures (5) outcomes measured by unbiased assessors (6) adequate response rate and refusers described (7) confidence intervals (CI) and subgroups analysis and (8) study subjects described. Each item in the tool is assigned a score of 1 with the total score ranging from 0 to 8, with more scores indicating a higher degree of quality.

### Statistical analysis

All data were analysed using OPEN META [Analyst] software version 10.12 [22] which is an open-source cross-platform database for advanced meta-analysis (developed by the Centre for Evidence Synthesis, Brown University, School of Public Health, RI, USA) and the funnel plots were generated using COMPREHENSIVE META-ANALYSIS software version 3.0(CMA 3.0 developed by a group of experts with funding from National Institute of Health in 2006, Englewood, NJ: Biostat). In all statistical analysis, the significance level was considered at a *P*-value <0.05. The overall prevalence of mental health outcomes among nurses was calculated using the random-effect model according to Der Simonian and Laird's approach at 95% confidence interval with Freeman turkey double arcsine transformation employed to stabilise the variance among studies [23,24].Heterogeneity testing was performed using the *I^2^* and Cochran’s Q test [25].We interpreted the *I^2^* statistic results as follows: 0 to 40% as not important, 30 to 60% as moderate heterogeneity 50 to 90% as substantial heterogeneity, 75 to 100% as considerable heterogeneity [26].Furthermore, a leave one out sensitivity analysis and subgroup analysis were performed to address the potential sources of heterogeneity. Publication bias among the included studies was addressed by funnel plot and eggers linear regression intercept [27].

## RESULTS

### Study characteristics

**Figure 1:**
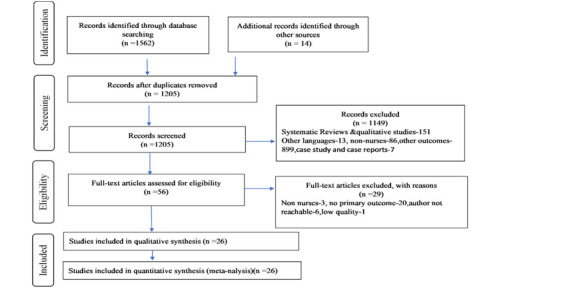
PRISMA flowchart depicting the selection process of included studies.

The initial search across different electronic databases and grey literature yielded 1576 citations. First, a total of 371 duplicate papers were excluded, accompanied by the removal of 1149 publications from the title/abstracts screening. Among the 56 full-text articles screened, 29 were not included. After screening the full text of 56 studies, 29 were excluded based on numerous factors; non-nurses as study participants (n = 3), the prevalence rate was not reported (n = 20) and the author not reachable (n = 6). Consequently, for further review, a total of 27 full-text articles matching the criteria for inclusion and exclusion were included **(****Figure 1****).** Among the 27 full-text articles included for systematic review, data of nurses concerning their mental health outcomes was made available to our study by requesting the corresponding author of 11 studies.

The essential attributes in the studies are displayed in **Table 1**. From March to August 2020, these studies were published with prevalence reports from different regions of the world namely; Asia (14), Europe (7), the Eastern Mediterranean Region (4) and the Americas (1). All the studies were cross-sectional, conducted among nurses in hospitals, via online web-based surveys while a single study was conducted nationally [33]. The majority of the reports focused on nurses as the primary sample (53.8%), while other health care professionals along with nurses accounted for the remainder (46.2%). The total sample size was 17 100 ranging from 45 [41] to 4692 [12]. Furthermore, for each of the mental ailments; anxiety, stress, depression, PTSD, insomnia, the percentage of nurses from the overall study sample (17,100) was 79.8%, 24.6%, 72.0%, 3.7%, 1.5%, respectively. Various validated scales with specific cut-off were used in our study such as; Generalised Anxiety Disorder, Zung’s Self-Rating Anxiety Scale, COVD-19 Anxiety Scale (CAS), The State-Trait Anxiety Inventory, Hospital Anxiety and Depression Scale, Stanford Acute Stress Reaction Questionnaire, Global stress Index (GSI), Self-reported Stressor and Incidence Questionnaire, Perceived Stress Scale, Hamilton Depression Rating Scale, Zung’s Self-Rating Depression Scale, Patient Health Questionnaire (PHQ-9), Depression Anxiety Stress Scale (DASS-21), Impact of Event Scale, PTSD Checklist-Civilian version and Pittsburgh Sleep Quality Index. However, no report has used formal psychiatric interviews to evaluate the presence of an actual illness or its severity. The participants' age ranged from 21-45 years and most were females (91.8%), while 8.2% were males. The majority of the sample was married (63.1%) and 36.9% were single.

**Table 1:** Characteristics of included studies

Primary author, month, year & country	Design& setting	Sample size (nurses)	Evaluating score	Prevalence of anxiety	Prevalence of stress	Prevalence of depression	PTSD	Insomnia	Significant risk factors of mental ailments*	Quality score†
Dal Bosco et al., May 2020, Brazil [9]	Cross-Sectional, Regional University Hospital	88	HAD-A&HAD-D>8	49% (43/88)	NA	25% (22/88)	NA	NA	Anxiety: 31-40 y, married women &working in critical sectors; Depression: 21 -30 y, single and women working in critical sectors-ICU, surgery and emergency	6
Darija Salopek-Ziha et al., June 2020, Croatia [10]	Cross-sectional, general hospital	71	DASS-21, Anxiety ≥ 10, Stress ≥ 15, Depression ≥ 10	24% (17/71)	21.1% (15/71)	26.8% (19/71)	NA	NA	NA	5
Eva-Maria Skoda et al., March 2020, Germany [28]	Cross-sectional study, nationwide	1511	GAD-7 ≥ 10	11.4% (172/1511)	NA	NA	NA	NA	NA	6
Zerbini et al.,2020, Germany [29]	Cross-sectional study,1 hospital	75	GAD-7 ≥ 10, PHQ-stress ≥ 5, PHQ-9 ≥ 10	16% (12/75)	45.3% (34/75)	29.3% (22/75)	NA	NA	Anxiety, stress, depression: caring for COVID-19 patients and working in COVID-19 wards.	5
Szepietowski et al., June 2020, Poland [30]	Cross-Sectional study, hospital	62	GAD-7 ≥ 5, PHQ-9 ≥ 10	GAD-7:46.7% (29/62)	NA	PHQ-9:21% (13/62)	NA	NA	NA	5
Bachilo et al., July 2020, Russia [31]	Cross-Sectional study, Hospital	139	GAD ≥ 5, PHQ ≥ 5	39.6% (55/139)	NA	50.4% (70/139)	NA	NA	NA	7
Giusti et al., July 2020, Italy [32]	Cross-Sectional Study, Hospital	86	DASS 21, Depression ≥ 4, Stress ≥ 7, IES>9, Anxiety ≥ 3	41.9% (36/86)	37.2% (32/86)	26.7% (23/86)	39.5% (34/86)	NA	Working in COVID-19 ward.	5
Shahrour et al., August 2020, Jordan [11]	Cross-sectional survey, hospitals.	448	SASRQ ≥ 56, ASD, GSI > 50-psycholoical distress	NA	ASD-64.1% (287/448) GSI-41.1% (184/448)	NA	NA	NA	Psychological distress, younger nurses, lower coping self-efficacy, higher ASD.	5
Naser et al., June 2020, Jordan [33]	Cross-sectional study, nationwide	151	GAD-7 ≥ 5, PHQ-9 ≥ 5	79.5% (120/151)	NA	80.8% (125/151)	NA	NA	NA	5
Deying et al., June 2020, China [34]	Cross-sectional study, two hospitals.	2014	SRA ≥ 50, SDS ≥ 23	41.4% (833/2014)	NA	43.6% (878/2014)	NA	NA	Anxiety and depression: low self-efficacy, resilience and social support.	8
Su Hong et al., July 2020, China [12]	Cross-sectional survey; 42 Government Hospitals.	4692	GAD-7 ≥ 10, PHQ-9 ≥ 10	8.1%, (379/4692)	NA	9.4% (442/4692)	NA	NA	Depression: Single, no support from family and hospital authority, being discriminated; Job-related stressors: High workload, being quarantined and impaired work ability.	7
Chen et al., July 2020, China [35]	Cross-sectional prospective survey, hospital.	92	SRISQ	NA	7-10 d-(68.5%), 63/92	NA	NA	NA	Working in isolation wards.	5
Yifang Zhou et al., June 2020 China [36]	Cross-sectional survey, community.	1614	PSQI ≥ 7	NA	NA	NA	NA	19.5% (314/1614)	Old age, Working in the emergency medical team.	5
Zhi-hao et al., May 2020, China [37]	Cross-sectional survey, hospital.	100	GAD-7 ≥ 4, PSQI ≥ 7, PHQ-9 ≥ 4	40% (40/100)	NA	46% (46/100)	NA	60% (60/100)	Depression: High anxiety. poor sleep quality and only child in the family.	5
Yu-Xin Zhan et al.,2020, China [38]	Cross-sectional, hospital.	2667	GAD-7 ≥ 5, CPSS ≥ 25, PHQ-9 ≥ 5	39.8% (1062/2667)	48.7% (1298/2667)	54.7% (1458/2667)	NA	NA	NA	7
Ying An et al., July 2020, China [39]	Cross-sectional survey, emergency department.	1103	PHQ-9 ≥ 5	NA	NA	43.7% (481/1103)	NA	NA	Depression: COVID Centre, current smokers, Emergency department nurses.	8
Ya-Xi Wang et al., May 2020, China [13]	Cross-sectional study, three hospitals.	202	PCL-C ≥ 38	NA	NA	NA	16.8% (34/202)	NA	PTSD: Female, negative coping, low job satisfaction.	6
Ruilin Li et al., June 2020, China [40]	Cross-sectional study, COVID hospital.	176	HAMA ≥ 7	77.3% (136/176)	NA	NA	NA	NA	Anxiety: long working hours, wearing protective equipment, female and great workload., working in COVID-19 designated hospitals.	6
Cuong Do Duy et al., July 2020, Vietnam [41]	Cross-sectional survey, COVID hospital.	45	DAS-21, Anxiety ≥ 3, Stress ≥ 4, Depression ≥ 4	11.1% (5/45)	2.2% (1/45)	11.1% (5/45)	NA	NA	NA	5
Saricam et al., July 2020, Turkey [42]	Cross-sectional study, hospital.	123	STAI ≥ 57	46.3% (57/123)	NA	NA	NA	NA	Anxiety: Advancing age and years of experience, working in wards and having a child; Working in pandemic and normal wards.	8
Lee et al., 2020, Singapore [43]	Cross-sectional study, tertiary hospital.	155	HADS-A > 10, HADS-D > 10	33.5% (52/155)	NA	31.6% (49/155)	NA	NA	Psychological distress: Multiple co-morbidities in staff, COVID care, quarantine order, redeployment outside normal professional boundaries.	8
Labrague et al.,2020, Philippines [44]	Cross-sectional study, hospitals.	325	CAS ≥ 9	37.8% (123/325)	NA	NA	NA	NA	Anxiety: Less social, organizational support and personal resilience.	8
Abdallah Badahdah et al.,2020, Oman [45]	Cross-sectional study,10 hospitals.	315	GAD-7 ≥ 10, PSS ≥ 24	11.4% (36/315)	58.7% (185/315)	NA	NA	NA	Stress: Females, COVID Centre	5
Pouralizadeh et al., August 2020, Iran [46]	Cross-sectional study, 25 hospitals.	441	GAD-7 ≥ 10, PHQ-9 ≥ 10	38.7% (171/441)	NA	37.4% (165/441)	NA	NA	Anxiety: Female nurses, COVID hospital, Suspected infection, lack of access to PPE; Depression-Chronic illness, suspected or positive cases, no access to personal protective equipment and female nurses	8
Wilson et al., July 2020, India [47]	Cross-sectional study, COVID-19 hospital.	55	GAD-7 ≥ 10, PSS ≥ 14, PHQ9 ≥ 10	21.8% (12/55)	80% (44/55)	14.5% (8/55)	NA	NA	Anxiety, Stress, Depression: female gender.	7
Chew et al., 2020, India and Singapore [48]	Cross-sectional study, tertiary hospitals.	350	DASS-21, IES, Anxiety > 7, Stress > 14, Depression > 9, PTSD > 24	9.4% (33/350)	3.7% (13/350)	7.1% (25/350)	6% (21/350)	NA	Anxiety, Stress, Depression, PTSD: Physical Symptoms (sore throat, breathlessness, cough, lethargy, myalgia, fever).	

HAD – Hospital Anxiety Depression Scale, HAM-A – Hamilton Anxiety Rating Scale, DASS – Depression Anxiety Stress Scale, GAD – Generalized Anxiety Disorder, PHQ – Patient Health Questionnaire, IES – Impact of Event Scale, SASRQ – Stanford Acute Stress Reaction Questionnaire, ASD – Acute Stress Disorder, GSI – Global Stress Index, SRISQ – Self Reported Stressor and Incidence Questionnaire, SRA – Self Rating Anxiety Scale, SDS – Self Rating Depression Scale, PSQI – Pittsburg Sleep Quality Index, PSS – Perceived Stress Scale, CAS – COVID-19 Anxiety Scale, PPE – personal protective equipment

*P < 0.05 or odds ratio >1.

**†**The Loney criteria [21].

### Quality evaluation

The methodological quality of the studies was assessed by two reviewers using Loney criteria [21]. Any discrepancy in the scoring between the two reviewers was resolved by mutual discussion and accord. The quality score ranged from 5-8 with 6 as the median score (interquartile range: 5-7) after the exclusion of a single study with a low-quality score of 4. Eventually, twenty-six studies with moderate or high quality were included for final analysis **(**Table S2 in the **Online Supplementary Document****).**

### Primary mental health outcomes

**Figure 2:**
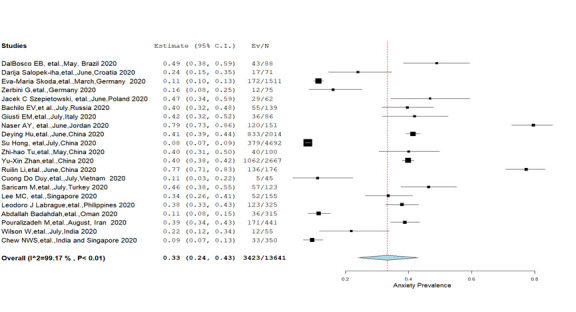
Pooled prevalence of anxiety among nurses (Q = 2467.18, df = 20).

**Anxiety:** A total of 21 studies [9,10,12,28-34,37,38,40-48] assessed the prevalence of anxiety among nurses and its severity level was appraised by eight studies [10,31,33,34,37,40,46,48]. The overall pooled prevalence of anxiety was 33% (95% CI = 24%-43%) with substantial heterogeneity (*I^2^* = 99.4%, *P* < 0.01) **(****Figure 2****).** Further, mild anxiety was more (24.8%) common compared to moderate (12.9%), severe (7.1%) and extreme severe anxiety (2%) estimated at a significant heterogeneity level. Moreover, no significant differences in the prevalence of anxiety were found in the subgroup analysis; males (27%), females (33.3%) and married (35.7%), single (31.5%) **(****Table 2****).** The regional analysis has shown that the prevalence of anxiety was more among nurses from the Eastern Mediterranean region (41.9%) compared to the European region (30.6%) and the combined prevalence reported from Western Pacific and South-East Asian regions (30.9%) **(****Table 3****).** The prevalence of mental health outcomes reported form Western Pacific and South-East Asian Regions were combined since our study included an individual study which was conducted across two different countries [48].

**Table 2:** Pooled prevalence of various subgroup categories of mental health outcomes among nurses

Mental health outcome	Variable	No. of studies	No. of participants	No. of positive cases	Χ-2 (*P*)	Heterogeneity					
**Estimate, % (confidence interval)**	***I^2^***	***P***	***Tau^2^***	***Q* (df)**
**Anxiety**	Mild anxiety	6	3195	832	596.4 (0.00001)	24.8 (13.7-37.9)	97.75	<0.001	0.03	222.06 (5)
	Moderate anxiety	7	3266	399		12.9 (8.2-18.4)	91.85	<0.001	0.01	73.856 (6)
	Severe anxiety	8	3442	230		7.1 (2.5-13.7)	96.53	<0.001	0.02	201.71 (7)
	Ex- severe anxiety	2	421	5		2 (0-7)	77.79	<0.001	0.01	4.50 (1)
	Males	4	206	34	3.8 (<0.06)	27 (4-58)	92.22	<0.001	0.09	38.57 (3)
	Females	4	5138	616		33.3 (10.4-61.6)	99.17	<0.001	0.08	359.42 (3)
	Married	4	3456	428	0.93 (0.34)	35.7 (11.8-64.1)	98.85	<0.02	0.08	262.93 (3)
	Single	4	1872	215		31.5 (8.5-60.7)	97.63	<0.02	0.09	126.57 (3)
**Stress**	Mild stress	2	421	10	6.4 (0.1)	2.9 (0.2-8)	68.66	<0.074	0.01	3.19 (1)
	Moderate stress	2	421	10		2.9 (0.2-8)	68.66	0.074	0.01	3.19 (1)
	Severe stress	2	421	6		2.3 (0-13.2)	90.92	<0.001	0.02	11.01 (1)
	Extreme severe stress	2	421	2		0.8 (0-5.3)	76.91	0.037	0.01	4.33 (1)
**Depression**	Mild depression	7	4269	1228	1524 (<0.0000)	23.8 (15.2-33.6)	97.56	<0.001	0.02	246.514 (6)
	Moderate depression	7	4269	445		11 (7.1-15.6)	93.24	<0.001	0.01	88.86 (6)
	Severe depression	7	4269	141		4 (1.6-7.3)	93.95	<0.001	0.01	99.167 (6)
	Extremely severe depression	4	1663	31		1.7 (0.4-3.6)	<0.001	0.002		10.614 (3)
	Males	4	276	69	11 (0.0009)	22.5 (2.8-51.6)	94.54	<0.001	0.08	54.98 (3)
	Females	4	6048	1042		27.9 (8.5-53)	99.55	<0.001	0.07	666.65 (3)
	Married	4	4090	692	2.7 (0.09)	26.9 (7-53.5)	99.42	<0.001	0.08	522.45 (3)
	Single	4	2218	412		28.4 (9.6-52.1)	98.56	<0.001	0.06	209.02 (3)
**PTSD**		3	638	89		18.6(4.8-38.3)	96.43	<0.001	0.04	56.02 (2)
**Insomnia**		2	1714	374		38.3(5.8-78.6)	98.56	<0.001	0.09	98.558 (1)

PTSD – posttraumatic stress disorder

*P < 0.05.

**Table 3:** Pooled estimate of mental health outcomes among nurses in different regions of the world

Region	Mental health outcomes	No. of studies	No. of participants	No. of Positives	Estimate, % (confidence interval)	Heterogeneity			
***I^2^***	***P-*value**	***Tau^2^***	***Q* (df)**
**European region**	Anxiety	7	2067	374	30.5 (16.7-46.3)	96.58	<0.001	0.05	175.68 (6)
	Stress	3	232	81	34.2 (21.2-48.6)	80.19	<0.001	0.01	10.01 (2)
	Depression	5	433	147	30.9 (20.4-42.5)	83.82	<0.001	0.02	24.73 (4)
**Western Pacific & South-East region**	Anxiety	10	10579	2720	30.9 (17.2-46.5)	99.53	<0.001	0.07	1931.52 (9)
	Stress	4	3165	1418	47.2 (14.7-81)	99.36	<0.001	0.13	467.66 (3)
	Depression	9	11181	3392	27.4 (13-44.7)	99.67	<0.001	0.07	2411.47 (8)
	PTSD	2	552	55	10.7 (2.5-23.5)	93.63	<0.001	0.01	15.70 (1)
	Insomnia	2	1714	374	38.3 (5.8-78.6)	98.56	<0.001	0.09	69.34 (1)
**Eastern Mediterranean region**	Anxiety	3	907	327	41.9 (10.7-77.3)	99.16	<0.001	0.11	273.25 (2)
	Stress	2	763	472	61.6 (56.4-66.8)	54.97	0.136	0.001	2.22 (1)
	Depression	2	592	290	61.2 (16.9-96.2)	99.04	<0.001	0.12	104.64 (1)

**Figure 3:**
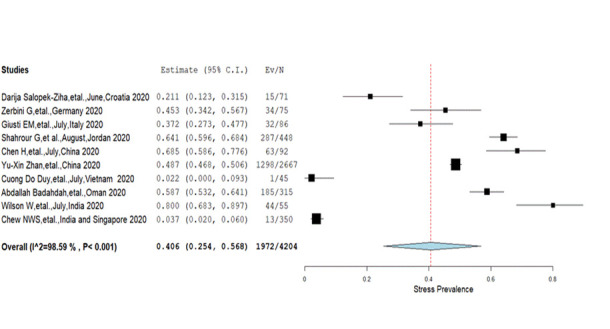
Pooled prevalence of stress among nurses (Q = 636.58, df = 9).

**Stress:** The overall prevalence of stress among nurses in the ten included studies [10,11,29,32,35,38,41,45,47,48] was 40.6% (25.4%-56.8%) with significant heterogeneity (*I^2^* = 98.6%, *P* < 0.001) **(****Figure 3****).** A comparative proportion of prevalence was found among different severity levels; mild (2.9%), moderate (2.9%), severe (2.3%) and extreme severe (0.8%) **(****Table 2****).** Besides, the Eastern Mediterranean region showed a higher prevalence of stress (61.6%) compared to Europe (34.2%) and the combined prevalence reported from Western Pacific and South-East Asian regions (47.2%) **(****Table 3****).**

**Figure 4:**
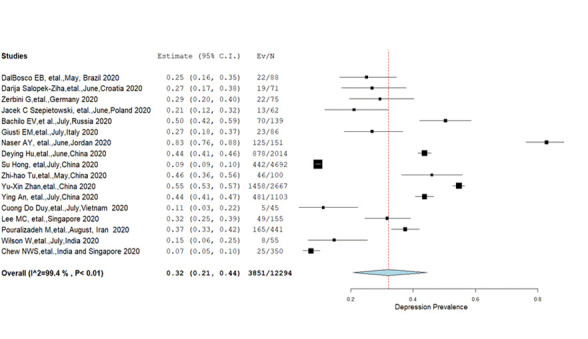
Pooled prevalence of depression among nurses (Q = 2659.04, df = 16).

**Depression:** Pooled prevalence of depression from 17 studies [9,10,12,29-34,37-39,41,43,46-48] was 32% (95% CI = 21%-44%) with significant heterogeneity (*I^2^* = 99.4%, *P* < 0.01) **(****Figure 4****).** Different estimates of pooled prevalence were found with respect to the degree of depression; mild (23.8%), moderate (11%), severe (4%) and highly severe (1.7%). While analysing subgroups; females had a significantly (*P* = 0.001) higher prevalence of depression (27.9%) than males (22.5%) but there were no significant differences (*P* = 0.09) in the prevalence rate between those who were married (26.9%) and single (28.4%) **(****Table 2****).** The pooled prevalence of depression (61.2%, 95% CI = 16.9%-96.2%) from the Eastern Mediterranean region (*P* < 0.00, *I^2^* = 99) reported a higher prevalence than in Europe (30.9%, 95% CI = 20.4%-42.5%) and combined prevalence from the Western Pacific and South-East Asian Regions (27.4%, CI = 13.0%-44.7%) **(****Table 3****).**

**PTSD and insomnia:** An imperceptible number of studies have evaluated PTSD which occurs as a secondary outcome due to stress (PTSD) [13,32,48]. The pooled prevalence of PTSD and insomnia at *I^2^* = 96.43 (*P* < 0.001) and *I^2^* = 98.56 (*P* < 0.001) was 18.6% (95% CI = 4.8%-38.3%) and 38.3% (95% CI = 5.8%-78.6%) respectively (**Table 2**).

**Risk factors for mental ailments:** The significant risk factors for anxiety, stress and depression as reported in different studies [9,11-13,29,34,36,37,39,40,42-48] were; direct care of COVID-19 patients, being a female, having low self-efficacy and resilience, poor social support and having physical symptoms (sore-throat, breathlessness, cough, lethargy, myalgia, fever). However specific risk factors for each of the mental outcomes evaluated from studies are as follows: i) anxiety: married women, having a child, aged 31-40 years, working in critical sectors, lack of access to personal protective equipment (PPE) and suspected infection; ii) stress: high workload, quarantine and impaired work ability; and iii) depression: being single, aged 21-30 years, having one child, having no support from family and hospital authority, being current smokers, having a chronic illness, poor sleep quality, high anxiety and redeployment outside professional boundaries. Moreover, explicit scrutiny was undertaken to identify the role of workplace on specific mental health outcomes. Anxiety, stress and depression were more common in nurses working in COVID-19 wards, COVID-19 designated hospital and critical sectors (Intensive Care Unit-ICU, surgery, emergency). However, a single study has reported that nurses working in normal wards were having more anxiety than those in ICU. A plausible reason attributed by the respective authors is that the safety environment and personal protection procedures in ICU enhanced the confidence of the nurses [42]. **(****Table 1****).**

### Publication bias and sensitivity analysis

We carried out an Egger’s publication bias plot [27] to detect the presence of publication bias which had an insignificant *P* value for all the primary mental health outcomes; anxiety (*t* = 0.23, *P* = 0.13), stress (*t* = 0.68, *P* = 0.52), depression (*t* = 0.52, *P* = 0.61), PTSD (*t* = 0.08, *P* = 0.47) indicating no substantial publication bias. In addition, a visual inspection of funnel plots for a Logit event rate of prevalence’s for mental health outcomes against corresponding standard error suggests evidence for the absence of publication bias **(**Figure S1, S2 and S3 in the **Online Supplementary Document****).** However, the publication bias was not addressed for the mental health outcome-insomnia, since there were only two studies.

A leave-one-out sensitivity analysis was performed for all the studies included under each of the specified mental health outcomes; anxiety (n = 21), depression (n = 17), stress (n = 10), PTSD (n = 3), insomnia (n = 2). Obviously no individual study had a considerable influence on the overall pooled prevalence for different mental health outcomes. Furthermore, the overall prevalence as reported in sensitivity analysis were; anxiety-33.2% (95% CI = 24.0%-43.0%), stress-40.4% (95% CI = 25.2%-56.6%), depression-32.6% (95% CI = 21.0%-45.5%), PTSD-19% (95% CI = 5.0%-38.0%), insomnia-38.4% (95% CI = 6.0%-78.6%).

## DISCUSSION

This systematic review and meta-analysis assessed the prevalence of mental health outcomes among nurses. Comparatively, higher rates of poor mental health outcomes namely, anxiety, stress, depression, PTSD and insomnia were observed among nurses from different parts of the world supporting our hypothesized statement. A high level of anxiety can be a precursor for other mental health outcomes, namely depression and insomnia [49] which has already been reported at moderate levels in our study. Depression bears the greatest burden of disability among mental and behavioural disorders [50]. This ultimately leads to many effects, including reduced job performance and quality, a greater risk of injuries at work, increased tardiness or absenteeism, impaired presenteeism, higher turnover rates, and a greater propensity for substance abuse [51-54]. It can be serious enough to lead to suicide in certain circumstances [55]. Studies conducted during the SARS outbreak also reported a similar proportion of depression, insomnia, emotional distress [56,57]. However, a lower prevalence rate was obtained for PTSD in our study as compared to a higher prevalence during SARS [56]. This discrepancy may be due to the inclusion of a significant number of studies conducted during the initial phase of COVID-19. Besides, this review highlighted the risk factors of mental health outcomes. There is no previous systematic review or meta-analysis that explored the prevalence of mental health outcomes among nurses during the COVID-19 pandemic.

Our study found that mental illness is common among nurses working in the frontline with COVID-19 patients. The prevalence of mental illness was not the same across different demographic groups and showed differences based on gender, marital status and geographic location. Multiple recent studies that explored the psychological status of health care professionals during the COVID-19 pandemic have emphasized the raised level of different types of pressures on nurses including higher exhaustion, higher stress perception, depressive symptoms, and lower job fulfilment compared to other health care professionals. This could be primarily due to the higher workload on them compared to their colleagues and the nature of their job responsibility that requires a prolonged duration of follow-up and direct contact with the patients.

Multiple variables were subjected to subgroup analysis to determine their role in the development of mental ailments. We did not find any statistically significant difference between subgroups based on gender or the marital status: males (27%) vs females (33.3%)) and (married (35.7%) vs single (31.5%). However, females had a significantly higher prevalence of depression (27.9%) than males (22.5%). At the same time, the prevalence of stress, PTSD and insomnia were not reported for subgroups in the studies included in the analysis. Our study identified the following risk factors for anxiety, stress and depression among nurses: caring for and being in direct contact with COVID-19 patients, being a female, low self-efficacy, resilience and social support and having physical symptoms (sore-throat, breathlessness, cough, lethargy, myalgia, fever). Comparatively, another study [58] reported that effective coping and self-adjustment strategies are essential for disaster rescuers to mitigate the burden of mental health problems; that they play a discerning role in responding efficiently and in a sustained and determined manner to the disaster. Furthermore, a similar study [59] have also reported that team cohesiveness is important to ensure that participants ask for support, alleviate pressure and develop coping strategies and mental resilience while responding to a new infectious disease outbreak. Similarly, lower self-efficacy, observed in our study as a risk factor for mental ailments is consistent with findings from earlier studies [60,61]. A recent report by the Department of Statistics in Canada has explored gender differences in mental health during the COVID-19 pandemic and reported that females are more likely to report worse mental health [62]. Another report from the United Kingdom has mentioned that the decline in the mental well-being of females was twice that for males. They emphasised the contribution of social factors in increasing the level of mental burden. Being socially active and having a larger social network before the pandemic was strongly associated with higher declines in the level of mental well-being after the pandemic. Females reported having more close friends before the pandemic than males, and higher loneliness than males after the pandemic [63]. Besides being a worker, woman having a family and other responsibility such as children were other important contributors to the negative impact on the mental well-being of females [64]. As seen in younger nurses in the study, an increased risk of developing psychological morbidity was also reported during the SARS outbreak [12]. This may be attributed to the insufficient expertise and inadequate preparation of younger nurses. However, the risk of anxiety was more pronounced in middle-aged nurses, which may be due to their perception of the family being getting infected by them. There was no significant difference between the nurses who were married and single in the prevalence of depression. On the contrary, several studies have reported that married nurses are having more serious depression than those who were single [65-67]. The inclusion of nurses from various parts of the world with cultural and geographical differences may be given as a potential reason for this.

Prevalence of mental illnesses was more common among nurses from the Eastern Mediterranean region compared to Europe and the combined prevalence reported from Western Pacific, South-East Asian region. A previous report by the United Nations highlighted that there is insufficient primary care in many countries in the Middle East region and that the health care system is fragmented. Besides, many factors such as unemployment, poverty, inadequate social safety nets, insufficiently responsive institutions and governance systems, and the economic shrinkage deeply impact the psychological and social status, and the general well-being of these populations [68]. Different studies from different regions have examined the negative impact of the COVID-19 pandemic on the relationship of different society segments. They have highlighted the negative influence on the relationship and communications between the members of the same family, colleagues at work and society as a whole [69-71].

High workload; cited as a contributing factor for negative mental health outcomes in most of the studies is intrinsically related to the global scarcity of nurses. The global shortage of nurses is 5.9 million, with the largest shortfall coming from low middle-income countries such as Pakistan, Nigeria, Indonesia and India. This is further substantiated by high stress reported from the South-East Asian region (47.2%) and the Eastern Mediterranean region (61.6%) in our review. To accelerate the response of the public health system during the pandemic as well as to attain sustainable development goals, there should be an increased investment for the nursing workforce in the subsequent years. An outlay of proximate US$10 per capita in low and lower-middle-income countries is required to augment the already existing nursing education [2].

### Strengths and limitations

Our systematic review and meta-analysis have many strengths:

1. To the best of our knowledge, this is the first systematic review and meta-analysis that has addressed the global prevalence of mental health outcomes among nurses during the COVID-19;

2. Data abstraction and quality assessment performed by two independent investigators which increases the robustness of our findings;

3. Freeman Tukey Double Arcsine transformation was employed to get pooled prevalence;

4. Included studies yielded data on nurses from different regions of the world which increase the generalisability of our estimates;

5. Sensitivity and subgroup analysis were done to explore the robustness of our estimates.

Nevertheless, our systematic review and meta-analysis have limitations:

1. Due to the lockdown policy implemented throughout the world, most of the included studies were cross-sectional web-based surveys, so that there could be a possibility of sampling bias;

2. The psychological status of nurses in the included studies was not evaluated before the pandemic. This restricted our ability to explore additional psychological burden on nurse due to the COVID-19 pandemic as we do not have data on their psychological status before it;

3. Substantial heterogeneity was identified among studies, which could be raised due to the difference in the assessment scales (variation in cut-offs scores) that were used across the studies to explore mental health outcomes;

4. Six studies were excluded as the authors of respective papers were not reachable;

5. Only research papers published in English were included, contributing to the lack of some studies from Asian countries in particular.

## CONCLUSION AND RECOMMENDATIONS

The current systematic review and meta-analysis have contributed empirical evidence on the deleterious effect of COVID-19 on the psychological health of nurses and in particular, manifested as anxiety, stress, depression, PTSD and insomnia. Consequently, health care organizations should prioritize the needs of nurses by providing various provisions such as short duty and adequate rest hours, sufficient protective supplies, online support services and due recognition to mitigate the vulnerability for poor mental health outcomes. The disaster preparedness plan envisaged by nations should have provisions to address the mental health of nurses which includes; regular screening for mental health issues, physical symptoms, promotion of coping strategies and resilience, targeted interventions to prevent PTSD. Furthermore, greater investment in addressing the global shortage of nurses should be given priority in national health policies especially in lower-middle-income countries that will make a substantial contribution to reacting to potential pandemics through decreased mental health demands on nurses. Moreover, attractive salary packages should be offered for preventing the emigration of nurses from lower-middle-income countries.

Our systematic review and meta-analysis suggest interventions to improve the psychological well-being of nurses during the COVID-19 pandemic. Counselling support should be provided through online workshops to enable nurses and other health care providers to cope with any potential psychological problems. Manpower should be increased and better resources allocation is recommended. Rotating nurses, providing flexible working schedules and encouraging nurses to use psychological support services are highly recommended. Further, studies evaluating the efficacy of mental health services to reduce the occurrence of poor mental health outcomes among nurses need to be carried out to ramp up the existing services.

## Additional material

Online Supplementary Document
