# Supplementary material for: Decline in the mental health of nurses across the globe during COVID-19: A systematic review and meta-analysis
Source: J Glob Health. 2021 Apr 10;11:05009. doi: 10.7189/jogh.11.05009 (PMC8053406; doi:10.7189/jogh.11.05009)
Supplement: Online Supplementary Document [file jogh-11-05009-s001.pdf]

Appendix S1: Search strategy

PubMed (Search hits=434)

Search: (Health personnel OR Nursing staff) AND (Covid-19) AND (mental health OR mental health disorders OR depression OR anxiety OR depressive disorder OR stress, psychological OR stress disorders OR Post-Traumatic OR sleep initiation and maintenance disorders) Filters: Journal Article, from 2020/3/11 - 2020/10/5

("health personnel"[MeSH Terms] OR ("health"[All Fields] AND "personnel"[All Fields]) OR "health personnel"[All Fields] OR ("nursing staff"[MeSH Terms] OR ("nursing"[All Fields] AND "staff"[All Fields]) OR "nursing staff"[All Fields])) AND ("severe acute respiratory syndrome coronavirus 2"[Supplementary Concept] OR "severe acute respiratory syndrome coronavirus 2"[All Fields] OR "ncov"[All Fields] OR "2019 ncov"[All Fields] OR "covid 19"[All Fields] OR "sars cov 2"[All Fields] OR (("coronavirus"[All Fields] OR "cov"[All Fields]) AND 2019/11/01:3000/12/31[Date - Publication])) AND ("mental health"[MeSH Terms] OR ("mental"[All Fields] AND "health"[All Fields]) OR "mental health"[All Fields] OR (("mental health"[MeSH Terms] OR ("mental"[All Fields] AND "health"[All Fields]) OR "mental health"[All Fields]) AND ("disease"[MeSH Terms] OR "disease"[All Fields] OR "disorder"[All Fields] OR "disorders"[All Fields] OR "disorder s"[All Fields] OR "disordes"[All Fields])) OR ("depressed"[All Fields] OR "depression"[MeSH Terms] OR "depression"[All Fields] OR "depressions"[All Fields] OR "depression s"[All Fields] OR "depressive disorder"[MeSH Terms] OR ("depressive"[All Fields] AND "disorder"[All Fields]) OR "depressive disorder"[All Fields] OR "depressivity"[All Fields] OR "depressive"[All Fields] OR "depressively"[All Fields] OR "depressiveness"[All Fields] OR "depressives"[All Fields]) OR ("anxiety"[MeSH Terms] OR "anxiety"[All Fields] OR "anxieties"[All Fields] OR

"anxiety s"[All Fields]) OR ("depressive disorder"[MeSH Terms] OR ("depressive"[All Fields] AND "disorder"[All Fields]) OR "depressive disorder"[All Fields]) OR ("stress, psychological"[MeSH Terms] OR ("stress"[All Fields] AND "psychological"[All Fields]) OR "psychological stress"[All Fields] OR ("stress"[All Fields] AND "psychological"[All Fields]) OR "stress psychological"[All Fields]) OR ("stress disorders, traumatic"[MeSH Terms] OR ("stress"[All Fields] AND "disorders"[All Fields] AND "traumatic"[All Fields]) OR "traumatic stress disorders"[All Fields] OR ("stress"[All Fields] AND "disorders"[All Fields]) OR "stress disorders"[All Fields]) OR "Post-Traumatic"[All Fields] OR ("sleep initiation and maintenance disorders"[MeSH Terms] OR ("sleep"[All Fields] AND "initiation"[All Fields] AND "maintenance"[All Fields] AND "disorders"[All Fields]) OR "sleep initiation and maintenance disorders"[All Fields]))

Web of Science Core collection (Search hits=126)

TITLE: (Nurse* AND (Covid 19 OR severe acute respiratory syndrome coronavirus 2))

Timespan: 2020.

Indexes: SCI-EXPANDED, CPCI-S, ESCI, CCR-EXPANDED.

Medline (Search hits=27)

TITLE: (Nurse* AND Covid 19)

Refined by: PUBLICATION YEARS: (2020) AND MESH HEADINGS: (NURSES) AND PUBLICATION YEARS: (2020)

Timespan: All years. Indexes: MEDLINE.

Psych info (search hits=110)

(Health personnel OR Nursing staff) AND (Covid-19 OR severe acute respiratory syndrome coronavirus 2 OR corona virus OR coronavirus infections) AND (mental health OR mental health disorders OR depression OR anxiety OR depressive disorder OR stress, psychological OR stress disorders, Post-Traumatic OR sleep initiation and maintenance disorders)

Narrowed by: Entered date: 2020-03-11 - 2020-10-05

Nursing And Allied Health database (Search hits=366)

Health personnel OR Nursing staff) AND (Covid-19 OR severe acute respiratory syndrome coronavirus 2 OR corona virus OR coronavirus infections) AND (mental health OR mental health disorders OR depression OR anxiety OR depressive disorder OR stress, psychological OR stress disorders, Post-Traumatic OR sleep initiation and maintenance disorders)

Narrowed by: Entered date: 2020-03-11 - 2020-10-05; Source type: Scholarly Journals

Science direct (search hits=160)

Title, abstract, keywords: Nurse AND Covid 19

Refined by: Research articles, short communications,

Publication year-2020

Coronavirus Research Database (Search hits=339)

(Health personnel OR Nursing staff) AND (Covid-19 OR severe acute respiratory syndrome coronavirus 2 OR corona virus OR coronavirus infections) AND (mental health OR mental health disorders OR depression OR anxiety OR depressive disorder OR stress, psychological OR stress disorders, Post-Traumatic OR sleep initiation and maintenance disorders)

Narrowed by: Entered date: 2020-03-11 - 2020-10-05; Document type: Article; Source type: Scholarly Journals

Table S1: Prisma statement and checklist

Section/topic	#	Checklist item	Page
**TITLE**			
Title	1	Identify the report as a systematic review, meta-analysis, or both.	1
**ABSTRACT**			
Structured summary	2	Provide a structured summary including, as applicable: background; objectives; data sources; study eligibility criteria, participants, and interventions; study appraisal and synthesis methods; results; limitations; conclusions and implications of key findings; systematic review registration number.	2,3
**INTRODUCTION**			
Rationale	3	Describe the rationale for the review in the context of what is already known.	3,4
Objectives	4	Provide an explicit statement of questions being addressed with reference to participants, interventions, comparisons, outcomes, and study design (PICOS).	5,6
**METHODS**			
Protocol and registration	5	Indicate if a review protocol exists, if and where it can be accessed (e.g., Web address), and, if available, provide registration information including registration number.	5
Eligibility criteria	6	Specify study characteristics (e.g., PICOS, length of follow-up) and report characteristics (e.g., years considered, language, publication status) used as criteria for eligibility, giving rationale.	6
Information sources	7	Describe all information sources (e.g., databases with dates of coverage, contact with study authors to identify additional studies) in the search and date last searched.	6

Search	8	Present full electronic search strategy for at least one database, including any limits used, such that it could be repeated.	6, Appendix S1
Study selection	9	State the process for selecting studies (i.e., screening, eligibility, included in systematic review, and, if applicable, included in the meta-analysis).	6,7
Data collection process	10	Describe method of data extraction from reports (e.g., piloted forms, independently, in duplicate) and any processes for obtaining and confirming data from investigators.	7
Data items	11	List and define all variables for which data were sought (e.g., PICOS, funding sources) and any assumptions and simplifications made.	6
Risk of bias in individual studies	12	Describe methods used for assessing risk of bias of individual studies (including specification of whether this was done at the study or outcome level), and how this information is to be used in any data synthesis.	7
Summary measures	13	State the principal summary measures	7
Risk of bias across studies	15	Specify any assessment of risk of bias (i.e. Newcastle-Ottawa Scale (NOS), that may affect the cumulative evidence.	7,8
Additional analyses	16	Describe methods of additional analyses (e.g., sensitivity or subgroup analyses, meta-regression), if done, indicating which were pre-specified.	8
**RESULTS**			
Study selection	17	Give numbers of studies screened, assessed for eligibility, and included in the review, with reasons for exclusions at each stage, ideally with a flow diagram.	8,9
Study characteristics	18	For each study, present characteristics for which data were extracted (e.g., study size, PICOS, follow-up period) and provide the citations.	9, Table1, Figure 1

Risk of bias within studies	19	Present data on risk of bias of each study and, if available, any outcome level assessment.	8, Table S1
Results of individual studies	20	For all outcomes considered (benefits or harms), present, for each study a summary data for each intervention group.	9,10, Table S2
Synthesis of results	21	Present results of study analysed.	10-11, Figure 2,3,4
Risk of bias across studies	22	Present results of any assessment of risk of bias across studies	10-11
Additional analysis	23	Give results of additional analyses, if done (e.g., sensitivity or subgroup analyses, meta-regression	12, Table 2,3
**DISCUSSION**			
Summary of evidence	24	Summarize the main findings including the strength of evidence for each main outcome; consider their relevance to key groups (e.g., healthcare providers, users, and policy [illegible]	13-15
Limitations	25	Discuss limitations at study and outcome level (e.g., risk of bias), and at review-level (e.g., incomplete retrieval of identified research, reporting bias).	16
Conclusions	26	Provide a general interpretation of the results in the context of other evidence, and implications for future research.	17-18
**FUNDING**			
Funding	27	Describe sources of funding for the systematic review and other support; role of funders for the systematic review.	1

Table S2: Methodological Quality assessment results of the eligible studies (The Loney Criteria)

First Author	Q1	Q2	Q3	Q4	Q5	Q6	Q7	Q8	Total Score
Eduardo Bassani Dal'Bosco	0	1	0	1	1	1	1	1	6
Darija Salopek-Ziha	0	0	0	1	1	1	1	1	5
Eva-Maria Skoda	1	0	1	1	1	1	0	1	6
Giulia Zerbini	0	0	0	1	1	1	1	1	5
Jacek C Szepieowski	0	0	0	1	1	1	1	1	5
Bachilo EV	1	1	1	1	1	1	1	0	7
Emmanuele Maria Giusti	0	0	1	1	1	1	0	1	5
Ghada Shahrour	0	0	1	1	1	1	0	1	5
Abdallah Y Naser	0	0	1	1	1	0	1	1	5
Deying Hu	1	1	1	1	1	1	1	1	8
Su Hong	1	1	1	1	0	1	1	1	7
Huijuan Chen	1	1	0	0	1	1	0	1	5
Yifang Zhou	0	0	1	1	1	0	1	1	5
Zhi-hao Tu	0	0	0	1	1	1	1		5
Yu-Xin Zhan	0	1	1	1	1	1	1	1	7
Ying An	1	1	1	1	1	1	1	1	8

Ruilin Li	0	0	1	1	1	1	1	1	6
Cuong Do Duy	0	0	0	1	1	1	1	1	5
Murat Saricam	1	1	1	1	1	1	1	1	8
Melvin CC Lee	1	1	1	1	1	1	1	1	8
Leodoro J Labrague	1	1	1	1	1	1	1	1	8
Abdallah Badahdah	1	1	1	1	1	0	0	0	5
Moluk Pouralizadeh	1	1	1	1	1	1	1	1	8
William Wilson	1	1	1	1	1	1	1	0	7
Chew NWS	1	1	1	1	1	1	0	1	7
Pramila Karki	0	0	0	1	1	1	1	0	4

Q1. Random sample or whole population Q2 Unbiased sampling frame Q3. Adequate sample size Q4. Standard measures Q5. Outcomes measured by unbiased assessors Q6. Adequate response rate and refusers described Q7. Confidence intervals (CI) and subgroups analysisQ8. Study subjects described.

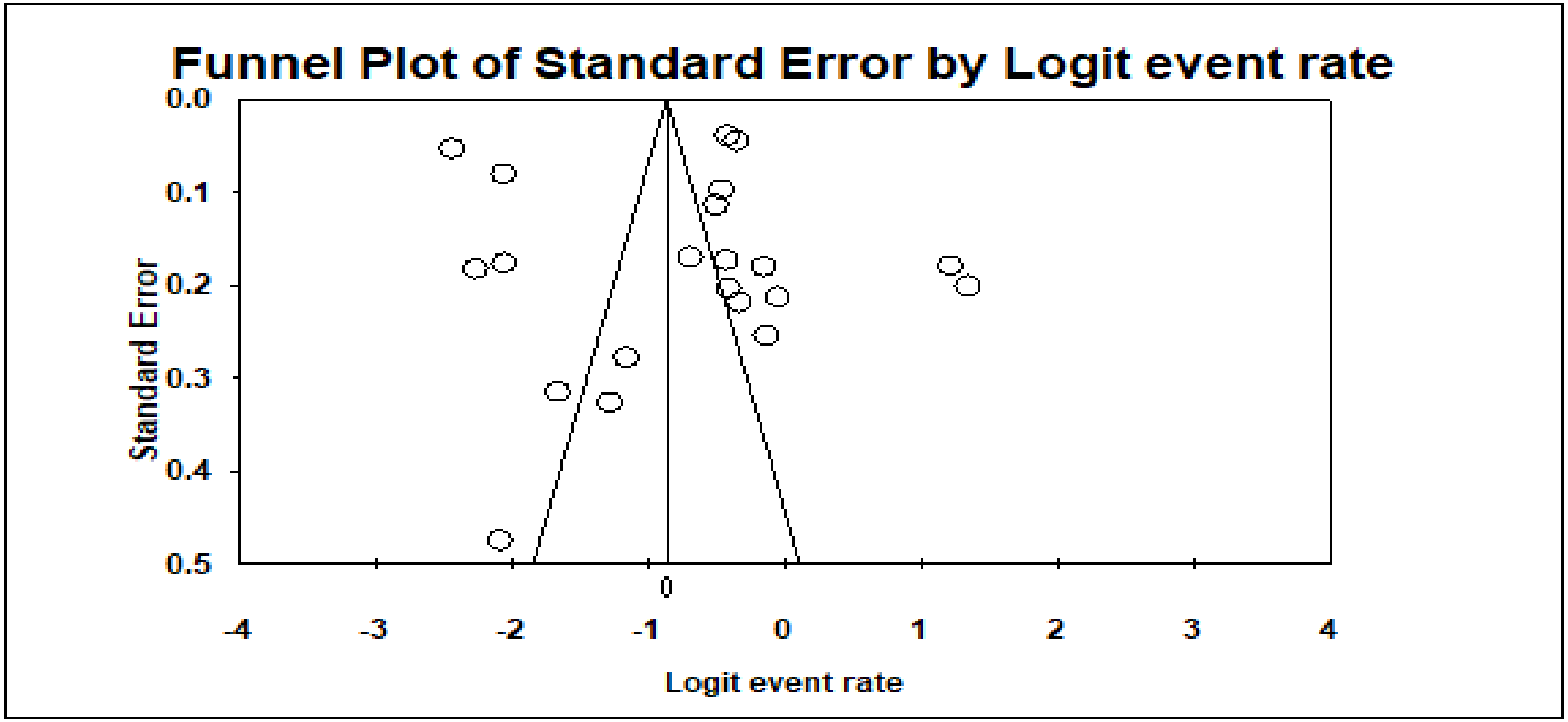

Figure S1: Funnel plot of 21 studies measuring anxiety.

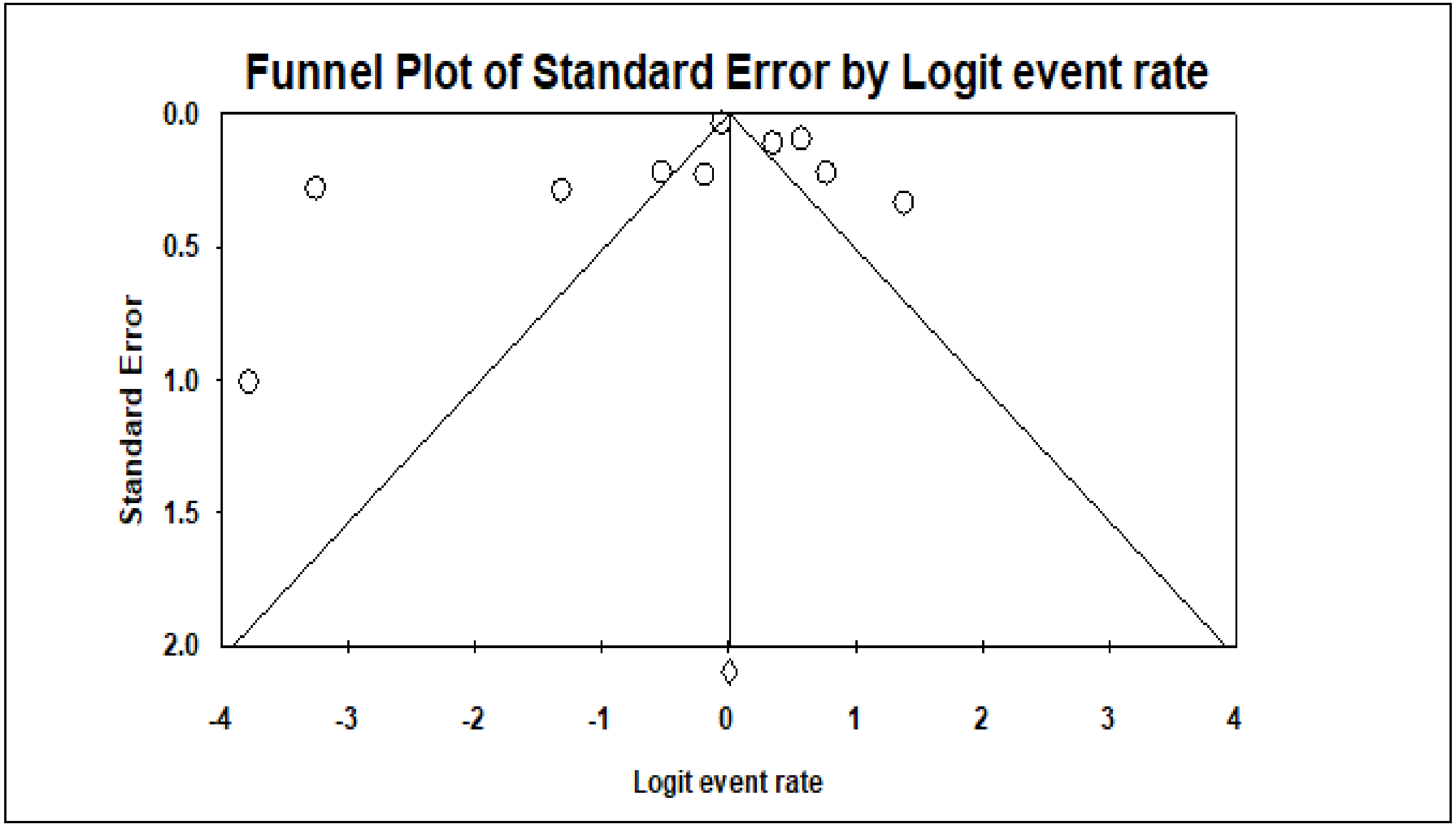

Figure S2: Funnel plot of 10 studies measuring stress.

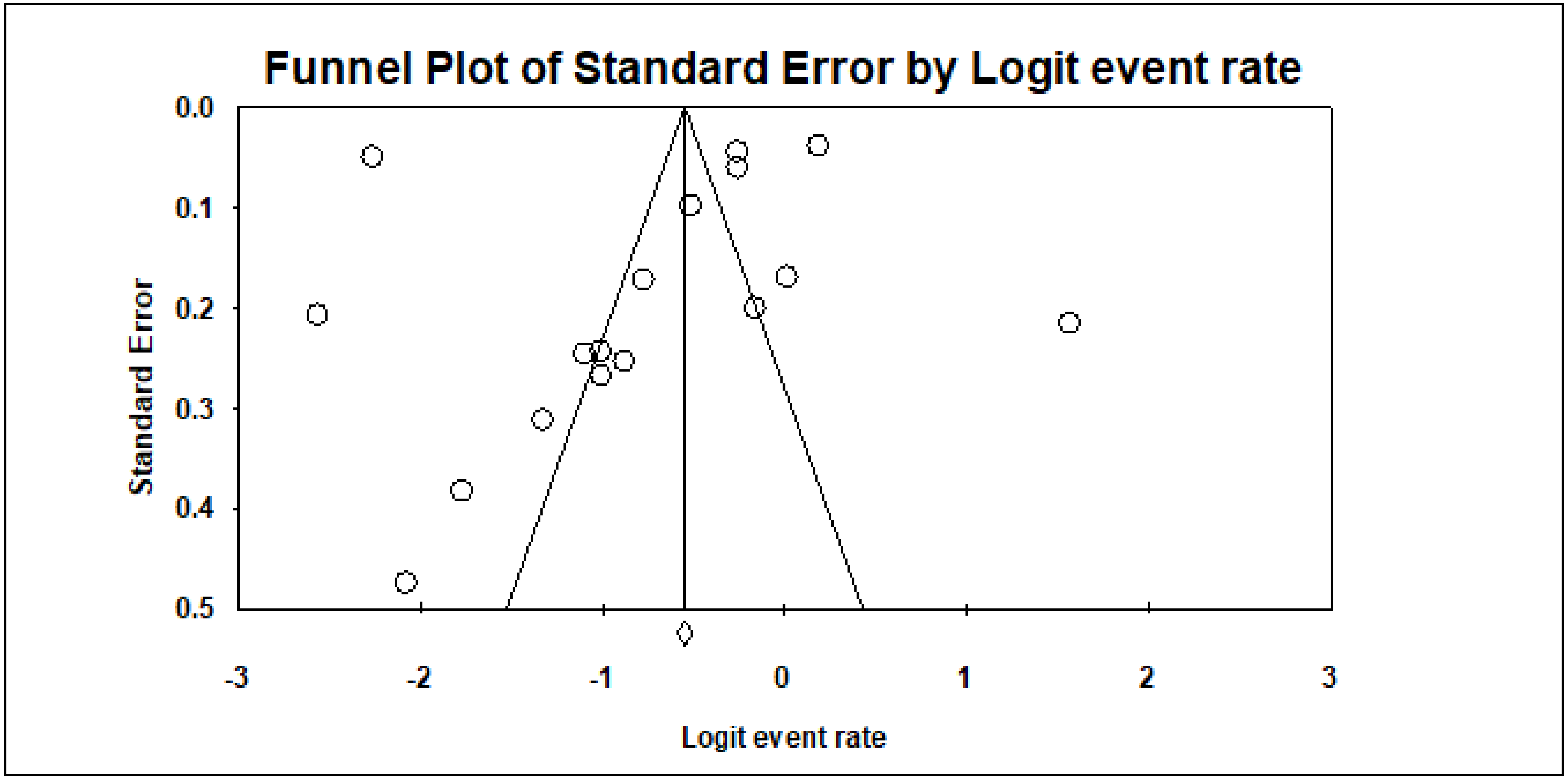

Figure S3: Funnel plot of 17 studies measuring depression
